# ﻿*Mosladadoensis* (Lamiaceae), a new species from the southern islands of South Korea

**DOI:** 10.3897/phytokeys.208.89552

**Published:** 2022-09-21

**Authors:** Hyun-Do Jang, Kwi-Kwan Jeong, Myoung-Ja Nam, Jun-Ho Song, Hye-Kyoung Moon, Hyeok Jae Choi

**Affiliations:** 1 Plant Resources Division, National Institute of Biological Resources, Incheon 22689, Republic of Korea Plant Resources Division, National Institute of Biological Resources Incheon Republic of Korea; 2 Yeosu Post Office, Korea Post (Citizen Researcher), Yeosu 59676, Republic of Korea Yeosu Post Office, Korea Post (Citizen Researcher) Yeosu Republic of Korea; 3 The Society for Korean Peninsula Plants (Citizen Researcher), Anyang 14069, Republic of Korea The Society for Korean Peninsula Plants (Citizen Researcher) Anyang Republic of Korea; 4 Herbal Medicine Resources Research Center, Korea Institute of Oriental Medicine, Naju 58245, Republic of Korea Herbal Medicine Resources Research Center, Korea Institute of Oriental Medicine Naju Republic of Korea; 5 Department of Biology, Kyung Hee University, Seoul 02447, Republic of Korea Kyung Hee University Seoul Republic of Korea; 6 Department of Biology and Chemistry, Changwon National University, Changwon 51140, Republic of Korea Changwon National University Changwon Republic of Korea

**Keywords:** Elsholtzieae, Korean endemic plant, morphology, phylogeny, taxonomy

## Abstract

*Mosladadoensis* (Lamiaceae), a new species from the southern islands of South Korea, is described and illustrated. The new species is morphologically similar to *M.chinensis*, but is distinguished from the latter by having two types of hairs on its stems, wider leaf blades, longer corolla length, and ellipsoid nutlets with a narrowly U-shaped extended area of abscission scar. *Mosladadoensis* is also distinguished from the Chinese narrow endemic *M.hangchouensis* by having an included pistil to the corolla, smaller ellipsoid nutlets, and later flowering and fruiting season. Phylogenetic analyses, based on two nuclear ribosomal (ETS, ITS) and three chloroplast (*rbc*L, *mat*K, *trn*L-F) DNA regions, confirmed that the new species was constructed as monophyletic, and that *M.dadoensis* and *M.hangchouensis* form a sister group with robust support. We hereby provide a detailed morphological description of *M.dadoensis* with its corresponding geographical distributions, and comparison tables of related taxa.

## ﻿Introduction

*Mosla* (Benth.) Buch.-Ham. ex Maxim. is a genus within the sixth largest family, Lamiaceae (the mint family). Although *Mosla* is a small genus of approximately 20 species, it is the second largest genus in the tribe Elsholtzieae (Wu and Li 1977a, b; Li and Hedge 1994; Harley et al. 2004). Elsholtzieae contains eight genera and roughly 70 species and is the smallest tribe within the most species-rich subfamily Nepetoideae (105 genera and about 3,400 spp.; Zhao et al. 2021). *Mosla* is mainly distributed in China, Japan, and Korea; however, *M.dianthera* (Buch.-Hamilt. ex Roxb.) Maxim. occurs in eastern Russia, the western Himalayas, and some Southeast Asian countries (Wu and Li 1977b; Li and Hedge 1994; Zhou et al. 1997; Govaerts et al. 2022). Phylogenetically, *Mosla* is nested within the eastern Asian *Mosla-Keiskea-Perilla* clade, and the monophyly of *Mosla* is strongly supported by previous morphological and taxonomic studies (Zhou 1995; Zhou et al. 1997; Li et al. 2017). Four fertile stamens are common in Elsholtzieae, and subequal or anterior pairs are normally longer, except in *Mosla*, which is characterized by two posterior fertile stamens (Harley et al. 2004; Kim 2018).

In Korea, four species of *Mosla* are recognized, namely *M.chinensis* Maxim., *M.dianthera*, *M.japonica* (Benth. ex Oliv.) Maxim., and *M.scabra* (Thunb.) C.W.Wu & H.W.Li (Seo and Park 2018; Korea National Arboretum 2020). The main diagnostic characteristics for species identification are the different leaf and calyx shapes. Leaves with a linear to linear-lanceolate shape are found only in *M.chinensis* and *M.japonica*, characterized by a subequal 5-toothed calyx. Although *M.scabra* and *M.dianthera* both have a 2-labiate calyx, the apex of the calyx lobe differs and is acute in *M.scabra* and obtuse in *M.dianthera* (Kim 2018).

During general floristic study in the southern part of Korea during October 2021, we found an unusual species which is restricted to the southern islands. This species is readily distinguished from previously known *Mosla* species in Korea by a considerably longer corolla. *M.chinensis* could be the closest ally, but the leaf shapes and flower features are significantly different. After a thorough literature survey and investigation of the relevant specimens, we designate *M.dadoensis* K.K.Jeong, M.J.Nam & H.J.Choi as a new species of *Mosla* from the southern islands of Korea. To clarify the systematic status of *M.dadoensis* we also conducted barcoding analysis based on nuclear ribosomal (nr) and chloroplast (cp) DNA regions, and observed detailed nutlet morphology, which is well known as a systematically important characteristic in Lamiaceae (Moon et al. 2009; Ryding 2010; Jeon et al. 2020). A detailed morphological description of *M.dadoensis* and its geographical distribution is also provided.

## ﻿Materials and methods

### ﻿Morphological characters

Morphological descriptions were based on specimens from the KB, KH (abbreviations are according to the Index Herbariorum [http://sweetgum.nybg.org/science/ih/]), and the herbarium of Changwon National University. Field surveys were also conducted from October 2021 to February 2022. Materials preserved in 70% ethanol were used for observation and measurement of floral parts. For quantitative characters, measurements were based on at least 50 samples.

### ﻿Microscopic analysis

For morphological observations and size measurements, the nutlets were first examined using a stereomicroscope (SM; Olympus SZX16, Olympus, Tokyo, Japan). Nutlet sizes were measured using at least 30 randomly chosen individuals from each species. Prior to scanning electron microscopic observations, all the dried nutlets were rehydrated overnight using the wetting agent Agepon (Agfa-Gevaert, Leverkusen, Germany) and distilled water (1:200) at 37–40 °C. The rehydrated materials were dehydrated through an ethanol series (50%, 70%, 90%, 95%, and 100%) at room temperature for 1 h each. The completely dehydrated materials were immersed in liquid carbon dioxide (CO_2_) for critical point-drying (CPD; SPI-13200J-AB, SPI Supplies, West Chester, PA, USA). For the micromorphological observations, selected nutlets were mounted on aluminum stubs using a double-sided adhesive conductive carbon disk (05073-BA, SPI Supplies, West Chester, PA, USA). Specimens were coated with gold using an ion-sputtering device (208HR, Cressington Scientific Instruments Ltd., Watford, UK), and then observed using a low-voltage field emission scanning electron microscope (FE-SEM; JSM-7600F, JEOL, Tokyo, Japan) at an accelerating voltage of 10 kV and a working distance of 8–10 mm (Song and Hong 2020).

### ﻿Phylogenetic analysis

To confirm the systematic placement of the putative new species within the genus *Mosla*, molecular phylogenetic analyses were conducted. The combined cpDNA dataset (*rbc*L, *mat*K, and *trn*L-*trn*F) and nrDNA dataset (ITS, ETS) used in Li et al. (2017) were employed with the addition of three individuals (*H.J.Choi 210923-001_1*–*3*) of the putative new species (Table 1). *Keiskeajaponica* Miq. was selected as the outgroup since it is a member of Elsholtzieae placed within the sister clade to *Mosla* (Li et al. 2017). Details of voucher information and GenBank accession numbers of the species used in this study are provided in Table 2.

**Table 1.** List of the primers used in phylogenetic analysis.

Fragment	Primer	Sequence 5' → 3’	Reference
ITS	ITS1	TCCGTAGGTGAACCTGCGG	White et al. (1990)
	ITS4	TCCTCCGCTTATTGATATGC	
ETS	ETS-B	ATAGAGCGCGTGAGTGGT	Baldwin and Markos (1998)
	18S-IGS	GAGACAAGCATATGACTACTG	Beardsley and Olmstead (2002)
*rbc*L	rbcL_1F	ATGTCACCACAAACAGAAAC	Fay et al. (1998)
	rbcL_724R	TCGCATGTACCTGCAGTAGC	
*mat*K	3F_Kim_F	CGTACAGTACTTTTGTGTTTA	K.J.Kim, pers. comm.
	1R_Kim_R	ACCCAGTCCATCTGGAAATCT	
*trn*L-F	B49317	CGAAATCGGTAGACGCTACG	Taberlet et al. (1991)
	A50272	ATTTGAACTGGTGACACGAG	

Total genomic DNA of *M.dadoensis* was extracted from silica gel-dried leaf materials using a DNeasy Plant Mini Kit (Qiagen Ltd., Crawley, West Sussex, UK). We conducted PCR with a ProFlex 96-Well PCR System (Applied Biosystems, Foster City, CA, USA). Each reaction mixture contained AccuPower PCR PreMix (Bioneer, Daejeon, South Korea), ca. 10 ng (1 μL) of genomic DNA, and 100 pM of primers in a total volume of 20 µL. Conditions included an initial denaturation at 95 °C for 5 min, followed by 40 amplification cycles comprising 95 °C for 30 sec, 50 °C for 30 sec, and 72 °C for 1 min, with a final extension at 72 °C for 5 min. After the PCR products were visualized on 2% agarose gels, they were treated with a MG PCR Purification kit (MGmed), and sequenced with the ABI 3730xl Analyzer, using the ABI BigDye Terminator v3.1 Cycle Sequencing Kits (Applied Biosystems, Foster City, CA, USA).

**Table 2.** List of voucher information and GenBank accessions of species used in this study.

Species	Voucher	ETS	ITS	*mat*K	*rbc*L	*trn*L-F
*Mosladadoensis 1*	H.J.Choi_210923_001_1	ON619797	ON033689	ON619803	ON619806	ON619800
*Mosladadoensis 2*	H.J.Choi_210923_001_2	ON619798	ON033690	ON619804	ON619807	ON619801
*Mosladadoensis 3*	H.J.Choi_210923_001_3	ON619799	ON033691	ON619805	ON619808	ON619802
*Moslacavaleriei*	PNLI20120445	KY552608	KY552540	KY624903	KY624972	KY625040
*Moslachinensis*	PNLI20120245	KY552609	KY552541	KY624904	KY624973	KY625041
*Mosladianthera*	PNLI20120248	KY552610	KY552542	KY624905	KY624974	KY625042
*Moslahangchouensis*	PNLI20120424-1	KY552611	KY552543	KY624906	KY624975	KY625043
*Moslajaponica*	PNLI20120416	KY552612	KY552544	KY624907	KY624976	KY625044
*Moslascabra*	PNLI20120427	KY552613	KY552545	KY624908	KY624977	KY625045
*Moslasoochouensis*	PNLI20120414	KY552614	KY552546	KY624909	KY624978	KY625046
*Moslatamdaoensis*	C-K-393	KY552615	KY552547	KY624910	KY624979	KY625047
*Keiskeajaponica*	PNLI20120049-1	KY552605	KY552537	KY624901	KY624969	KY625037

Phylogenetic analyses were conducted using maximum likelihood (ML). The obtained sequences were aligned using MAFFT with Geneious Prime 2019.2.3 (Biomatters Ltd., Auckland, NZ). To assess the confidence of the phylogenetic relationships, a bootstrap test was conducted with 1,000 replications for the ML analysis. Kimura’s three-parameter model (Kimura 1980) was selected as the substitution model.

## ﻿Results and discussion

### ﻿Taxonomic treatment

#### ﻿ Mosla dadoensis

Taxon classificationPlantaeLamiales Lamiaceae

K.K.Jeong, M.J.Nam & H.J.Choi sp. nov.

30C7A5A0-EF34-5431-957F-1151501EA086

urn:lsid:ipni.org:names:77305495-1

**Figure 1.**
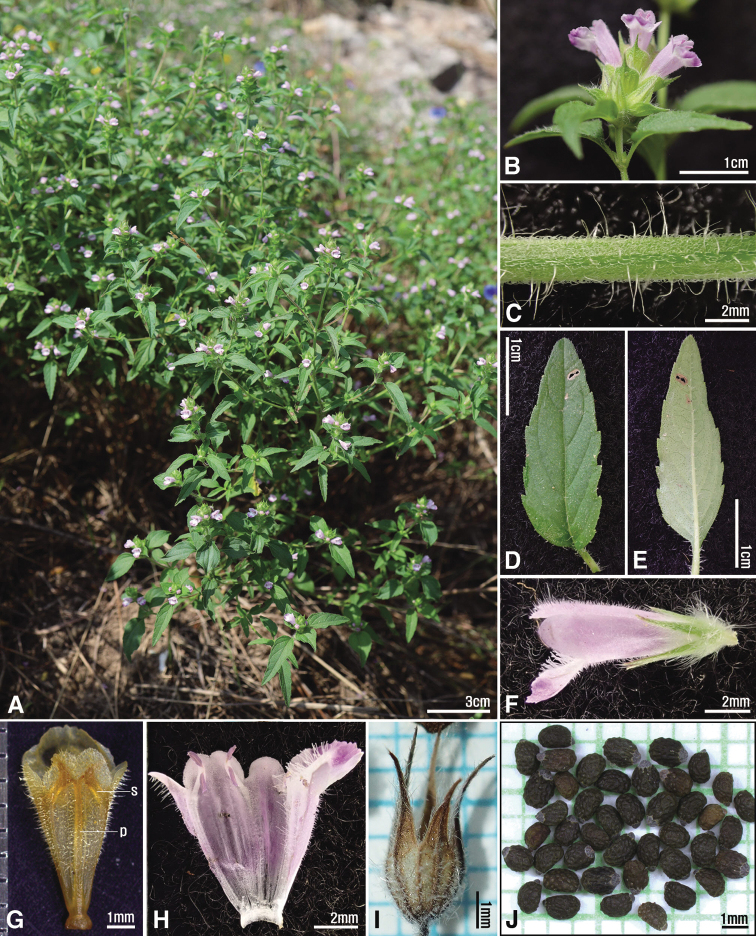
*Mosladadoensis***A** habit **B** raceme **C** stem **D, E** leaf (**D** adaxial **E** abaxial) **F** flower **G, H** corolla (**S** stamen **P** pistil) **I** calyx **J** seed. Photos from *H.J.Choi 210923-001* (**A–H**) and *H.J.Choi 211025-001* (**I, J**).

**Figure 2.**
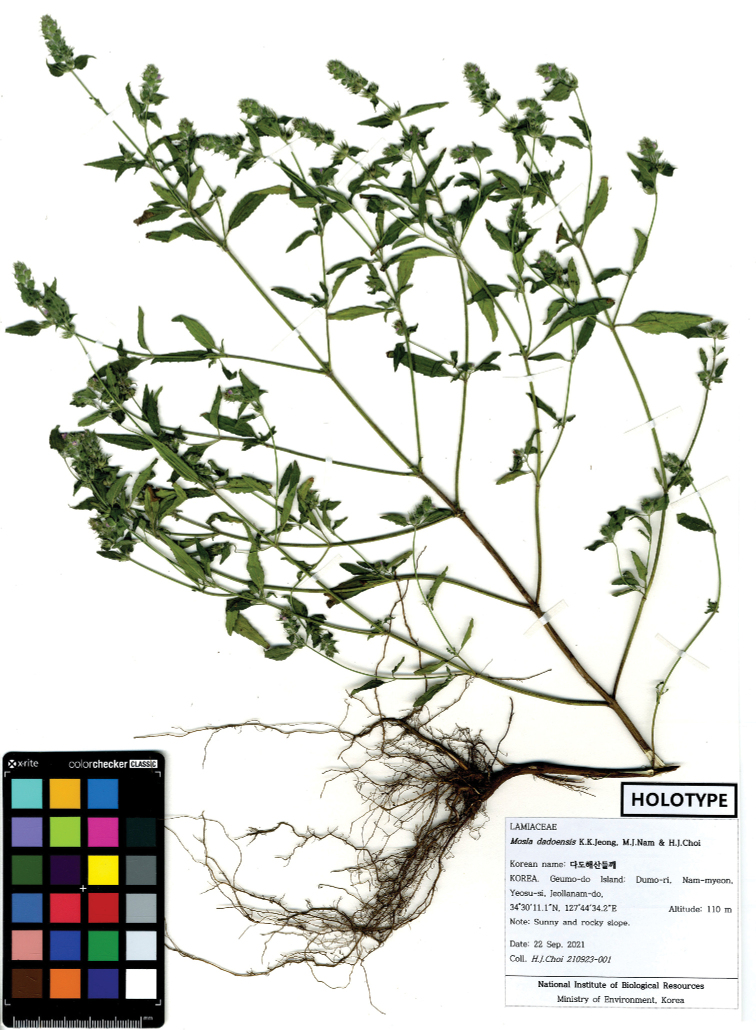
Photograph of the holotype of *Mosladadoensis*.

**Figure 3.**
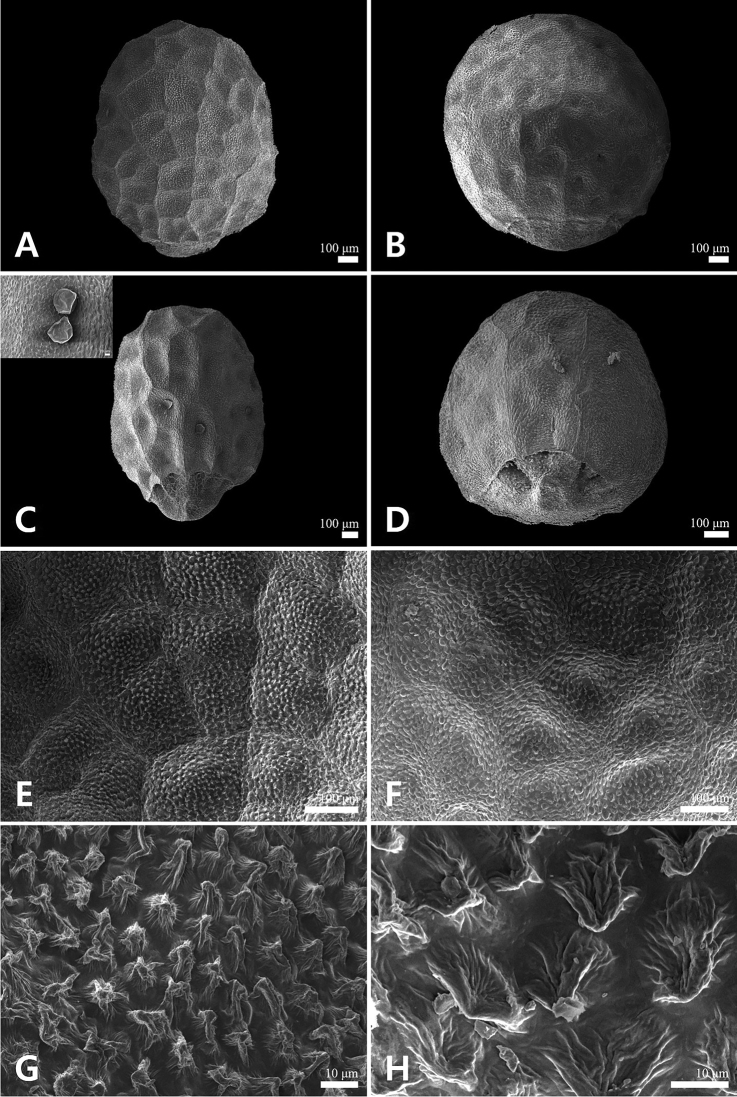
Scanning electron microscope micrographs of nutlets in *Mosladadoensis* (**A, C, E, G**) and *M.chinensis* (**B, D, F, H**) **A, B** abaxial **C, D** adaxial (**C** small picture showing gland) **E, F** primary sculpture pattern **G, H** secondary sculpture pattern (micropapillate).

**Figure 4.**
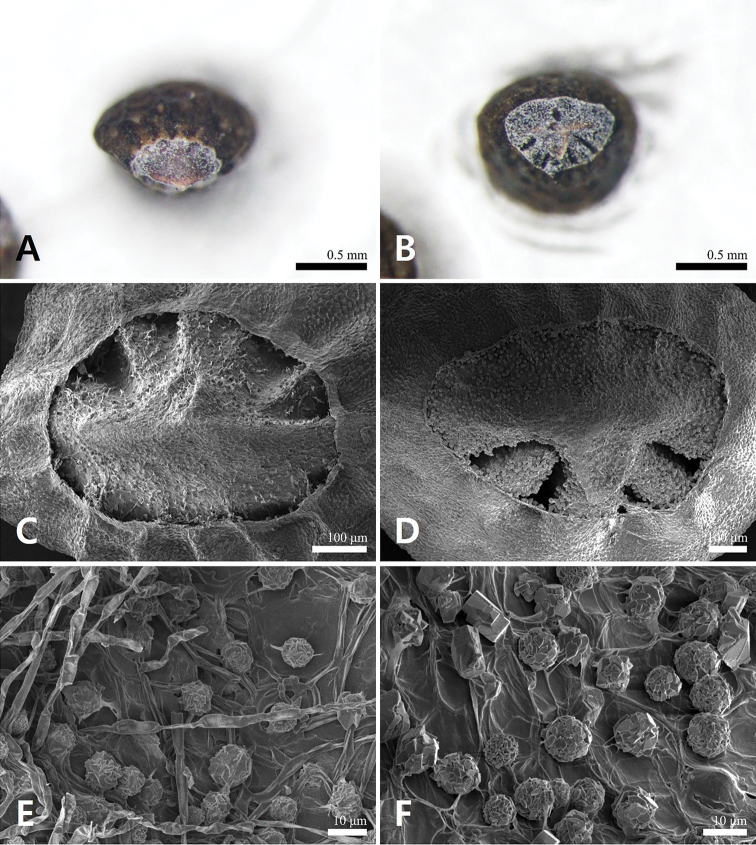
Stereo- and scanning electron microscope micrographs of abscission scar in *Mosladadoensis* (**A, C, E**) and *M.chinensis* (**B, D, F**).

Figs 1 , 2 , 3A, C, E, G , 4A, C, E

##### Diagnosis.

This new species is morphologically similar to *M.chinensis*, but is easily distinguished from the latter by having two types of hairs on its stems, wider leaf blades, longer corolla length, and ellipsoid nutlets with a narrowly U-shaped extended area of abscission scar.

##### Type.

Korea. Jeonnam: Yeosu-si, Geumo-do Isl., 34°30'11.1"N, 127°44'34.2"E, elev. 110 m, 22 Sep 2021 [fl], *H.J.Choi 210923-001* [Holotype: KB (Fig. 2); Isotypes: CWNU, KB, KH, KIOM, KSM].

##### Description.

Herbs annual, aromatic. Stems 10–60 cm tall, many branched from base, densely pubescent with white recurved hairs and densely to moderately intermixed with white villous, with impressed glands. Leaves petiolate; petiole 2–5 mm long, pubescent with white villous; blades narrowly lanceolate to lance-ovate, 1–3 cm × 4–10 mm, sparsely pubescent, dotted with impressed glands, adaxially olive green, abaxially gray, base cuneate, margin remotely serrate, apex acute. Racemes terminal, 1–2.5 cm, bracts overlapping, circular-obovate, 5–7 × 4–5 mm, margin ciliate, apex caudate. Pedicel pubescent. Calyx campanulate, ca. 5 × 3 mm, dilated after anthesis, subequally 5-toothed; teeth subulate, ca. 2/3 to 3/4 as long as calyx tube. Corolla slightly 2-labiate, pale purple, ca. 1.5 times longer than bracts, 8–9 mm long, pubescent outside, pubescent with long white villous on lower lip inside; upper lip straight, emarginate; lower lip 3-lobed, middle lobe largest, slightly recurved. Stamens 4, included (non-exserted); filaments shorter than anthers; anthers linear, cells divergent, ca. 2 mm long, connectives distinct. Pistil included; sigma bifid. Nutlets brown to blackish-brown, ellipsoid, 1.2–1.6 × 0.9–1.3 mm, glabrous or sparsely pubescent with gland, pitted with deep depressions, abscission scar basal position, elliptic, extended, extended area narrowly U-shaped at the ventral side, ratio of abscission scar / nutlet diameter 0.51–0.53, primary sculpture outline of cells isodiametric, tetragonal to hexagonal, anticlinal walls straight, raised, thin, periclinal walls concave, secondary sculpture micropapillate.

##### Phenology.

Flowering and fruiting from August to November.

##### Distribution and habitat.

**Figure 5.**
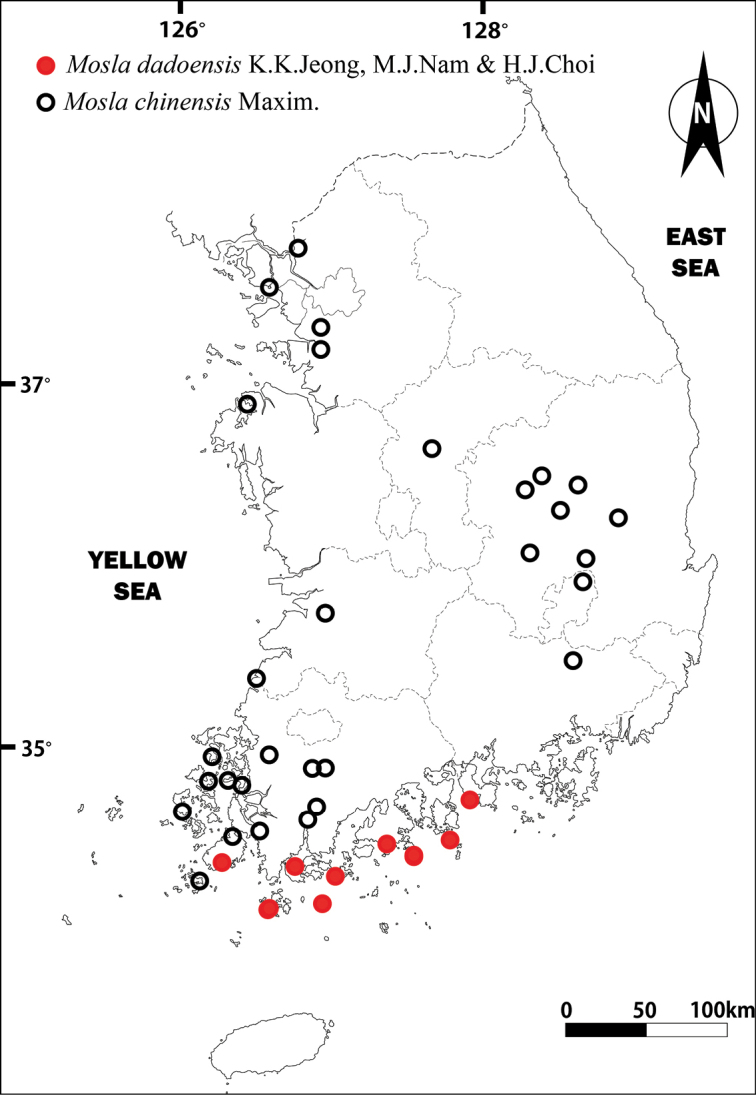
Distribution map of *Mosladadoensis* and *M.chinensis* in Korea.

Endemic to southern coastal regions of Korea (Fig. 5). Open rocky area near the coast; at altitudes of 8–500 m.

##### Etymology.

The specific epithet, “*dadoensis*”, is based on the name of location, the Dadohae southern coastal region of Korea, where *Mosladadoensis* was discovered.

##### Vernacular name.

The Korean name of the new species is “Da-do-hae-san-deul-kkae (다도해산들깨)”.

##### Morphological assessment.

*Mosladadoensis* is morphologically similar to *M.chinensis*, from which it is clearly differentiated by the hairs on its stems [white recurved hairs and intermixed with white villous (Fig. 1B) vs. white recurved hairs only], shape and size of leaf blades [narrowly lanceolate to lance-ovate, 1–3 cm × 4–10 mm (Figs 1D, E) vs. linear to linear-lanceolate, 1–5 cm × 1.3–4 mm], length of corolla [8–9 mm (Figs 1G, H) vs. 5–6 mm], and shape of nutlets [ellipsoid with narrowly U-shaped extended area of abscission scar (Figs 1J, 3A, C, 4A, C) vs. globose to subglobose with widely U-shaped extended area of abscission scar (Figs 3B, D, 4B, D)]. *Mosladadoensis* is also distinguished from *M.chinensis* by its distinctive tetragonal to hexagonal nutlet surface cells with straight and thin anticlinal walls (Table 3; Fig. 3). In addition, this new species is morphologically similar to Chinese narrow endemic *M.hangchouensis* Matsuda. However, it is easily distinguished by its length of corolla [8–9 mm and ca. twice as long as calyx (Fig. 1F) vs. ca. 10 mm and ca. three times longer than calyx], relative length of pistil to the corolla [included (Figs 1B, F, G) vs. clearly exserted], shape and size of nutlets [ellipsoid, 0.9–1.3 mm in diam. (Figs 1J, 3A, C) vs. globose to subglobose, ca. 2.1 mm in diam], and later flowering and fruiting season (August to November vs. June to September). The major characters of the new species are compared to those of the related *M.chinensis* and *M.hangchouensis* in Table 3.

**Table 3.** Comparison of major characters of *Mosladadoensis*, *M.chinensis*, and *M.hangchouensis* (*: data from Li and Hedge 1994; Zhou et al. 1997; Ge and Chang 2001).

Character		*M.dadoensis*	*M.chinensis*	M.hangchouensis*
Habitat		open rocky area along the coast	grassy slope, forest edge, wet land	sunny side of hill peak, forest edge, and under forest along the coast
Plant	height (cm)	10–60	10–40	20–120
Stem	trichome	densely pubescent with white recurved hairs and moderately intermixed with white villous	densely pubescent with white recurved hairs	pubescent, brown glandular sometime intermixed with spreading pilose hairs
Leaf blade	shape	lanceolate to lance-ovate	linear to linear-lanceolate	lanceolate
	size	1–3 cm × 4–10 mm	1–5 cm × 1.3–4 mm	1.5–4.2 cm × 5–13 mm
Corolla	length (mm)	8–9	5–6	ca. 10
	length ratio of corolla/calyx	ca. 2.0	ca. 1.5	ca. 3.0
Pistil	relative length to corolla	included	included	clearly exserted
Nutlet	shape	ellipsoid	globose to subglobose	globose to subglobose
	diameter (mm)	0.9–1.3	1.0–1.2	ca. 2.1
	extended area of abscission scar	narrowly U-shaped at the ventral side	widely U-shaped at the ventral side	widely U-shaped at the ventral side
	ratio of abscission scar/nutlet diameter	0.51–0.53	0.61–0.69	*NA*
	outline of surface cell	tetragonal to hexagonal	rounded	tetragonal to hexagonal
	anticlinal walls of surface	straight, thin	curved, thick	straight, thin
Flowering and fruiting		August to November	June to October	June to September

##### Phylogenetic analysis.

**Figure 6.**
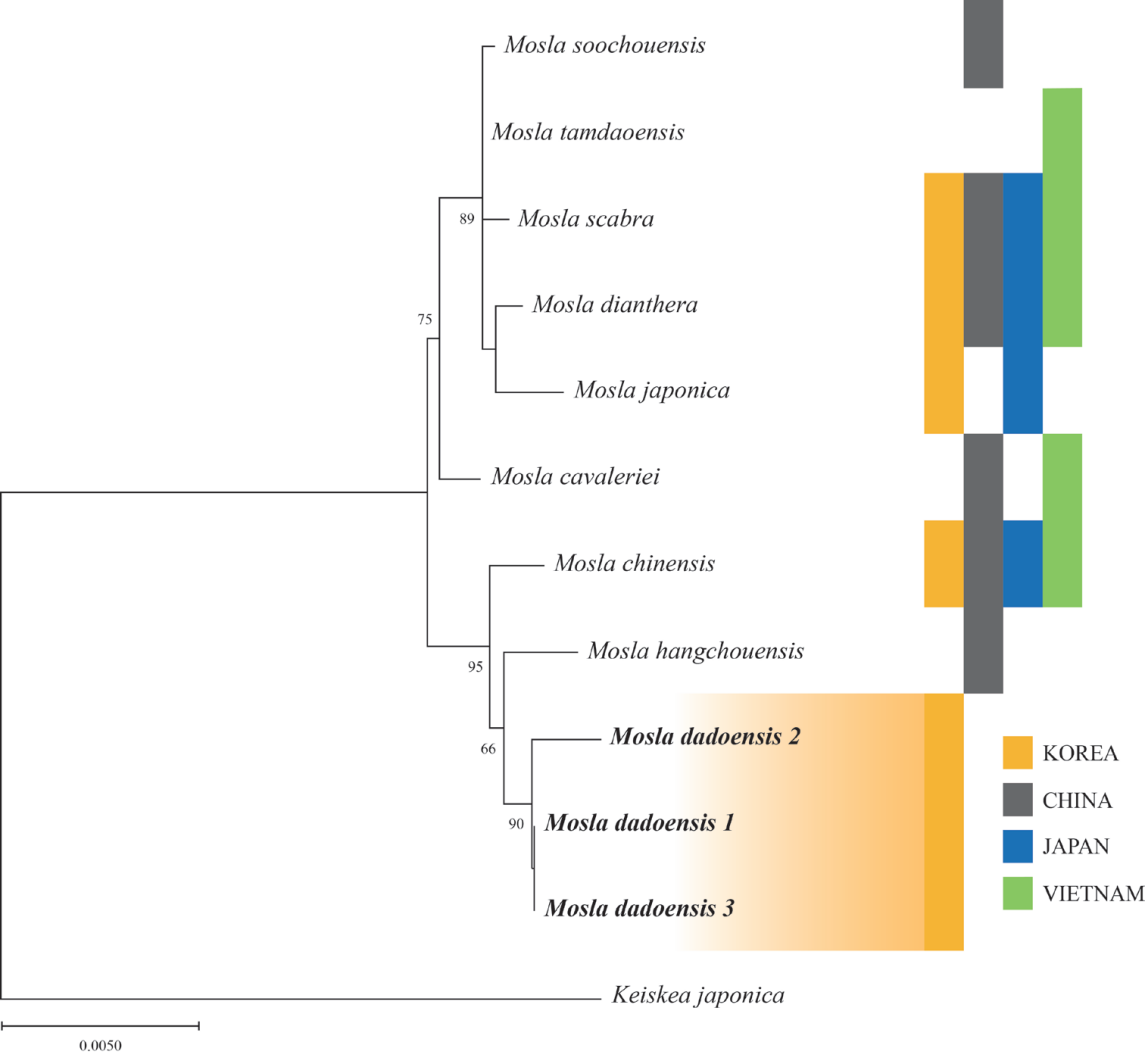
Phylogenetic tree of *Mosladadoensis* and related taxa based on concatenated alignments of two nrDNA (ITS, ETS) and three cpDNA regions (*rbc*L, *mat*K, *trn*L-F). The numbers above branches are bootstrap values (BS > 50%) used in the maximum likelihood method. Distribution information was obtained from Plants of the World Online (https://powo.science.kew.org).

The combined dataset has 12 aligned sequences comprising 2,910 bp (609 bp for ITS, 371 bp for ETS, 439 bp for *rbc*L, 736 bp for *mat*K, and 755 bp for *trn*L-F), of which 102 occupied variable positions (3.51%). Our phylogenetic tree (Fig. 6) revealed a similar topology to that obtained in the previous study (Li et al. 2017). *Mosla* species were constructed as monophyletic, and *M.dadoensis* was classified as a clade independent from other members of *Mosla* on the ML tree. *M.dadoensis* was distinguished from *M.chinensis*, a related species distributed in China and Korea. Instead, the tree is shown to form a clade closer to *M.dadoensis* in Korea and *M.hangchouensis* in China (Fig. 6).

##### Additional specimens examined.

*Mosladadoensis* (*Paratypes*): **Korea: Jeonnam**: Yeosu-si, Geumo-do Isl., 34°30'11.1"N, 127°44'34.2"E, elev. 110 m, 25 Oct 2021 (fr), *H.J.Choi 211025-001* (CWNU); Goheung-gun (Naro-do Isl.), Bongrae-myeon, Jangpo-san, 34°25'25.46"N, 127°30'26.90"E, elev. 307 m, 26 Feb 2022, *K.K.Jeong s.n.* (CWNU); Goheung-gun (Naro-do Isl.), Bongrae-myeon, Bongrae-san, 34°25'45.51"N, 127°30'56.77"E, elev. 150 m, 26 Feb 2022, *K.K.Jeong s.n.* (CWNU); Jindo-gun, Yeogui-san, 34°23'41.91"N, 126°14'21.00"E, elev. 296 m, 27 Feb 2022, *K.K.Jeong s.n.* (CWNU); Jindo-gun, Imhoe-myeon, Namdong-ri, Hanbok-san, 34°22'18.1"N, 126°9'42.7"E, elev. 96 m, 9 Oct 2013 (fl, fr), *JJP7102* (KB); Goheung-gun, Dohwa-myeon, 34°29'14.01"N, 127°19'26.06"E, elev. 8 m, 3 Mar 2022, *K.K.Jeong s.n.* (CWNU); Goheung-gun, Dohwa-myeon, 34°26'42.11"N, 127°20'06.30"E, elev. 39 m, 3 Mar 2022, *K.K.Jeong s.n.* (CWNU); Wando-gun, Bogil-myeon, Jeokja-bong, 3 Oct 2003 (fr), *B.Y.Sun et al. s.n.* (KB); Wando-gun, Bogil-myeon, Yesong-ri, Geokja-bong, 34°32'38.88"N, 126°55'39.32"E, elev. 303 m, 24 Oct 2013 (fr), *kjs 130042* (KB); Wando-gun, Wando-eup, Daeya-ri, 34°22'12.22"N, 126°40'56.03"E, elev. 505 m, 11 Aug 2014, *Y.H.Cho & H.J.Na 140811107* (KB); Wando-gun, Bogil-myeon, Buhwang-ri, 34.128717N, 126.535047E, elev. 50 m, 7 Oct 2017, *WR-171007-075* (KH); Wando-gun, Bogil-myeon, Yesong-ri, Gyeokjabong, 34°08'15.50"N, 126°33'32.90"E, elev. 148 m, 7 Nov 2009 (fr), *HNHM-2010-0355* (KH); Wando-gun, Cheongsan-myeon, Cheonggye-ri, 34.159905N, 126.897922E, elev. 80 m, 8 Sep 2017 (fl, fr), *WR-170908-003* (KH); Wando-gun, Saengil-myeon, Bongseon-ri, 7 Sep 2003 (fl), *Lee.Y.H. 030062* (KH). **Gyeongnam**: Namhae-gun (Namhae-do Isl.), Nam-myeon, Eungbong-san, 34°43'40.24"N, 127°53'15.65"E, elev. 268 m, 1 Mar 2022, *K.K.Jeong s.n.* (CWNU).

*Moslachinensis*: **Korea: Gyeonggi**: Anyang-si, Dongan-gu, Bisan-dong, 37°25'23."N, 126°57'34.4"E, elev. 235 m, (fr), *PWK-133* (KH); Suwon-si, Gwonseon-gu, Homaesil-dong, Chilbo-san, 37°15'40.39"N, 126°55'47.4"E, elev. 84 m, 24 Sep 2009 (fr), *NIBRVP0000209769* (KB); Incheon-si, Ganghwa-gun, Gilsang-myeon, Donggeom-ri, Donggeom-do Isl., 37°35'25.2"N, 126°31'2.7"E, elev. 63 m, 8 Sep 2012, *NIBRVP0000400499* (KB); Paju-si, Tanhyeon-myeon, Bupheung-ri, 37°46'02.4"N, 126°41'19.4"E, elev. 100 m, 30 Aug 2006, *VP-NAPI-376034-053* (KB). **Chungbuk**: Jeungpyeong-gun, Jeungpyeong-eup, Jwagu-san, 36°42'41.8"N, 127°39'39.2"E, elev. 500 m, 25 Aug 2011 (fl), *Geumbuk-203* (KH). **Chungnam**: Seosan-si, Daesan-eup, Ungdo-ri, 36°55'04.4"N, 126°22'24.8"E, elev. 0 m, 15 Aug 2012, *DJUIDC20120154* (KH). **Jeonbuk**: Gimje-si, Dojang-dong, Hwang-san, 35°46'35"N, 126°56'30.1"E, elev. 12 m, 27 Aug 2011, *357014-0420* (KB). **Jeonnam**: Haenam-gun, Hwangsan-myeon, Wonho-ri, Hakdong village, 34°34'15.14"N, 126°29'2.22"E, elev. 3 m, 17 Sep 2008 (fl), *ParkSH81875* (KH); Jindo-gun, Jodo-myeon, Sinyuk-ri, Hajo-do Isl., Sinjeon beach, 34°17'21.5"N, 126°01'88.1"E, elev. 39 m, 6 Sep 2011, *HS110899* (KH); Sinan-gun, Docho-myeon, Oryu-ri, Near Simok Sandbeach, 34.725447N, 125.908671E, elev. 30 m, 24 Oct 2007, *WR-071024-170* (KH); Sinan-gun, Docho-myeon, Oryu-ri, Near Simok Sandbeach, 34.725447N, 125.908671E, elev. 30 m, 24 Oct 2007, *NAM-071024-199* (KH); Yeonggwang-gun, Hongnong-eup, Gyema-ri, Gamami beach, 11 Sep 2012, *P126974* (KH); Hwasun-gun, Doam-myeon, Daecho-ri, Cheonbulsan, Unjusa, 34°55'27.3"N, 126°52'14.0"E, elev. 109 m, 6 Sep 2009, *SGU 0940* (KH); Gangjin-gun, Byeongyeong-myeon, Jiro-ri, Suin-san, 34°42'56.9'N, 126°50'18.5"E, elev. 174 m, 12 Aug 2014 (fl), *HNHM-D-140197* (KH); Sinan-gun, Aphae-myeon, Songgong-ri, Songgong-san wetland, 34.844604N, 126.252203E, elev. 25 m, 19 Sep 2007 (fl), *WR-070919-255* (KH); Jindo-gun, Gunnae-myeon, Geumseong-ri, 34°32'54.1"N, 126°17'38.6"E, elev. 30 m, 14 Sep 2005 (fr), *ESJeon 52851* (KH); Naju-si, Dado-myeon, Masan-ri, Bulhoe-sa Temple, 34°55'15.7"N, 126°53'35.8"E, elev. 126 m, 14 Sep 2005, *ESJeon 52829* (KH); Hampyeong-gun, Hakgyo-myeon, Gokchang-ri, 35.026751°N, 126.570272°E, elev. 100 m, 9 Sep 2012 (fl), *WR-20120909-044* (KH); Sinan-gun, Amtae-myeon, Songgok-ri, Amtae-do Isl., 34°50'20.5"N, 126°8'35.9"E, elev. 13 m, 10 Oct 2019, *YLJLVP0000006165* (KB); Gangjin-gun, Gundong-myeon, Pungdong-ri, Seongjak-gol, 34°30'48.45"N, 126°40'49.90"E, elev. 294 m, 23 Sep 2010, *C201009-0117* (KB); Sinan-gun, Aphae-myeon, Janggam-ri, 34°49'3.9"N, 126°20'58.1"E, elev. 14 m, 4 Oct 2012 (fl), *KOSPVP0000256241* (KB); Jindo-gun, Gunnae-myeon, Dunjeon-ri, Geumgol-san, 34°32'24.8"N, 126°17'39.2"E, elev. 81 m, 27 Oct 2013 (fl), *KOSPVP0000291190* (KB); Sinan-gun, Jeungdo-myeon, Jeungdong-ri, Gubunpo, Gwakdae-bong to Bunpo reservoir, 28 Sep 1997 (fl), *EN97CUB404* (KB). **Gyeongbuk**: Sangju-si, Jungdong-myeon, Hoesang-ri, Hwanggeum-san, 36°27'55.3"N, 128°16'34.2"E°, elev. 210 m, 9 Sep 2012 (fl), *KTPSA-2012076* (KH); Daegu-si, Dong-gu, Jimyo-dong, 35°56'25.09"N, 128°39'49.04"E, elev. 202 m, 21 Aug 2013 (fl), *DJUIDC2013-212* (KH); Yecheon-gun, Jibo-myeon, Amcheon-ri, 36°33'06.00"N, 128°27'11.09"E, elev. 11 m, 7 Sep 2011 (fl), *Nakdong-1632* (KH); Gyeongbuk, Sangju-si, Jungdong-myeon, Hoesang-ri, 36°27'54.9"N, 128°16'37.0"E, elev. 236 m, 8 Sep 2012, *NAPI2012-0153* (KH); Cheongsong-gun, Hyeonseo-myeon, Hwamok-ri, 36°16'24.4"N, 128°52'22.1"E, elev. 387 m, 3 Aug 2018, *NIBRVP0000703391* (KB); Gunwi-gun, Bugye-myeon, Changpyeong-ri, San 100, 36°4'56.36"N, 128°41'34.59"E, elev. 225 m, 27 Sep 2019 (fr), *NIBRVP0000756907* (KB); Andong-si, Iljik-myeon, Wonho-ri, Jaam-san, 36°29'54.08"N, 128°40'37.14"E, elev. 302 m, 21 Aug 2017 (fl), *NIBRVP0000632258* (KB); Gunwi-gun, Bugye-myeon, Changpyeong-ri, 36°4'59.04"N, 128°41'35.97"E, elev. 220m, 29 Aug 2019, *NIBRVP0000754852* (KB); Uiseong-gun, Bian-myeon, Jarak-ri, Haemang-san, 36°22'52.31"N, 128°31'3.79"E, elev. 202 m, 3 Oct 2017 (fr), *NIBRVP0000643724* (KB); Gimcheon-si, Nam-myeon, Busang-ri, Geumo-san, San 168-7, 36°3'58.8"N, 128°16'34.1"E, elev. 220 m, 20 Sep 2015 (fl), *NIBRVP0000585241* (KB); Gumi-si, Namtong-dong, Geumo-san, Peak to Beopseong temple, 36°5'52.5"N, 128°19'46"E, elev. 250 m, 5 Oct 2015 (fr), *NIBRVP0000586707* (KB). **Gyeongnam**: Milyang-si, Muan-myeon, Garye-ri, Yeongchwi-san, 35°29'57.40"N, 128°35'02.20"E, elev. 201 m, 14 Sep 2009 (fr), *HNHM-2009-0392* (KH).

## Supplementary Material

XML Treatment for Mosla dadoensis
